# Droplet Microfluidics for Chip-Based Diagnostics

**DOI:** 10.3390/s141223283

**Published:** 2014-12-05

**Authors:** Karan V. I. S. Kaler, Ravi Prakash

**Affiliations:** Department of Electrical and Computer Engineering, University of Calgary, 2500 University Drive NW, Calgary, AB-T2N 1N4, Canada; E-Mail: rprakash@ucalgary.ca

**Keywords:** droplet microfluidics, electro-actuation, dielectrophoresis, electrowetting, electrostatic force, nucleic acid, PCR, qPCR, immunoassays, proteomics, photomultiplier tube

## Abstract

Droplet microfluidics (DMF) is a fluidic handling technology that enables precision control over dispensing and subsequent manipulation of droplets in the volume range of microliters to picoliters, on a micro-fabricated device. There are several different droplet actuation methods, all of which can generate external stimuli, to either actively or passively control the shape and positioning of fluidic droplets over patterned substrates. In this review article, we focus on the operation and utility of electro-actuation-based DMF devices, which utilize one or more micro-/nano-patterned substrates to facilitate electric field-based handling of chemical and/or biological samples. The underlying theory of DMF actuations, device fabrication methods and integration of optical and opto-electronic detectors is discussed in this review. Example applications of such electro-actuation-based DMF devices have also been included, illustrating the various actuation methods and their utility in conducting chip-based laboratory and clinical diagnostic assays.

## 1. Introduction

The term ‘droplet microfluidics (DMF)’ is generally associated with the handling of fluidic sample droplets, in the volume range of microliters (10^−6^ L) to picoliters (10^−12^ L). This droplet handling capability is achieved through a variety of methods by utilizing micro-fabricated and/or micro-machined structures in the order of a few hundred micrometers to nanometers. A key attribute of the DMF actuation methodology is the rapid and automated handling of fluidic samples, in the form of discrete droplets that are dispensed, transported, merged, split and temperature cycled on patterned substrates [1–7]. At microscopic scales, capillary forces dominate over the inertial effects [1,4], and therefore, the majority of droplet actuation methods rely on controlling the interfacial energy at the solid-liquid, liquid-liquid interfaces. There are other methods that rely on the sufficient induction of body forces within the liquid mass, juxtaposed with interfacial forces to create stronger actuation regimes [2,3,6].

**Figure 1.**
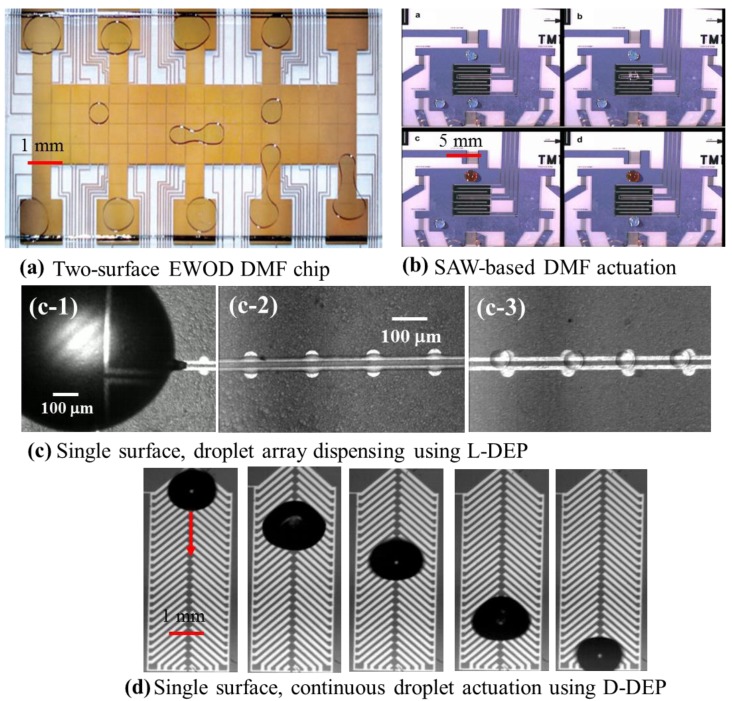
(**a**) Droplet actuations on a two-surface electrowetting on dielectric (EWOD) device (reproduced with permission from [7]); (**b**) droplet actuation using SAW (reproduced with permission from [6]); (**c**) rapid droplet array dispensing using liquid dielectrophoresis (L-DEP) (reproduced with permission from [49]); (**d**) continuous droplet transport using droplet-DEP (D-DEP) (reproduced with permission from [3]).

The approach towards such droplet manipulation in DMF can be broadly classified as passive and active droplet actuation schemes [1]. Amongst the passive methods, thermal [8,9], chemical [1,9] interfacial [10] and topographical effects [11] are most commonly used for microfluidic applications. However, such schemes tend to be rather slow and often not suitable for large array-based applications, where high throughput is crucial for efficient device performance. In active droplet actuation methods, droplets are dispensed and transported using active and switchable field effects, which result in faster and more efficient maneuvering during targeted applications. Such active actuation methods include: electro-actuation [1–5,12], magnetic actuation [13,14], acoustic [15], surface acoustic [6,15] and optical liquid actuation [16,17]. Many of the active droplet actuation methods have been utilized towards the demonstration of chemical and biological assays, including polymerase chain reaction (PCR) [18–22] and immunoassays [23–26]. This review will primarily focus on the electro-actuation of droplets and their diagnostic applications [9,27,28]. The electrostatic field generates the driving force behind any form of electro-actuation for droplets. However, electro-actuation techniques are generally categorized based on the way the electric field is manifested on the liquid droplet. Electrowetting (EW) [1,4,5,7] and dielectrophoresis (DEP) [2,3,28,29] are two very popular electro-actuation methods for droplets. The EW actuation technique relies on controlling the interfacial equilibrium of a sessile droplet by applying an external electric field [1,30,31]. Electrowetting-based droplet actuation can be achieved either on a single micro-patterned surface [4,32] or with the droplet sandwiched between two patterned substrates [5,7,33]. In either case, EW-based droplet actuation requires active switching of multiple planar metal electrodes to facilitate sustained droplet manipulation, and as a result, such droplet microfluidic techniques are termed as digital microfluidics [5,34–38]. EW can also be achieved using light/optics (opto-electrowetting) by incorporating a patterned photoconductive material/substrate, with the electrowetting electrodes patterned on top [16,17]. Such opto-electrowetting techniques can achieve active electrode switching during droplet actuations using a tailored optical source, which can significantly reduce the electrical overhead requirement at the expense of the associated optical components. Dielectrophoresis (DEP) is an electrokinetic phenomenon, which arises on a dielectric as a ponderomotive body force, when it is subjected to a spatially non-uniform electric field [39,40]. Liquid-DEP (L-DEP) has been leveraged as a rapid droplet dispensing technique, capable of dispensing arrays of precision homogeneous [2,24,41], multiphase/emulsion [42,43], suspensions/colloids [28,44] and variable volume droplets [45] in the volume range of sub-microliters to picoliters. DEP has also been exploited for maneuvering functionalized micro-particles, cells and lipid vesicles for sample preparation and other bio-diagnostic applications [43,46–48]. Another unique electro-actuation technique, originally investigated as single-phase electrostatic droplet actuation [12,28], requires coplanar arrangement of herringbone-shaped electrodes. The method, also termed droplet-DEP (D-DEP), relies on creating an asymmetric and periodic deformation cycle in a droplet, oscillating the droplet continuously in a uni-directional fashion [3,12]. This review highlights various bio-diagnostic applications that leverage such electro-actuation methods. Figure 1 shows the structural features and images of droplet actuation over four DMF micro-chips, exploiting surface acoustic wave (SAW), EW, L-DEP and D-DEP electro-actuation methods.

## 2. Theoretical Background

The theoretical analysis of both DEP and EW techniques has previously been reviewed in [1,2,29,31,50]. There are several factors that influence droplet actuation in DMF, and they can be generally divided into two categories: driving forces and resistive forces. The resistive forces are comprised of capillary force and viscous drag force, which opposes the droplet actuation. For electro-actuation-based DMF technology, the driving force is essentially generated as a result of an externally-applied electric field. Theoretical methods for estimating the driving forces generated as a result of an externally applied electrostatic field have been analyzed and reported in several works [30,33,41,50]. Early analyses were based on a thermodynamic approach using the Young–Lippmann equation [1,51],

(1) $$\text{Cos}\theta =\text{Cos}\theta_{0}+\frac{{ɛ}_{0}{ɛ}_{\text{r}}V^{2}}{2\gamma \text{d}}$$

where θ and θ_0_ are static contact angles of the droplet, with and without the applied voltage; ε_r_ is the relative dielectric permittivity; ε_0_ is the permittivity of free space; V is the applied voltage; γ is the fluidic surface tension; and d is the dielectric thickness. In this analysis, droplet actuation was assumed to occur entirely due to the capillary pressure as a result of the asymmetric contact angles across the droplet. This capillary force can be derived from:

(2) $$L_{\gamma }\left(\text{Cos}\theta -\text{Cos}\theta_{0}\right)=\frac{{ɛ}_{0}{ɛ}_{\text{r}}LV^{2}}{2\text{d}}$$

where L is the length of the contact line overlapping the energized electrode. This approach towards understanding electrowetting on dielectric (EWOD) has numerous references in the literature [1,4,5]. The driving force is a result of electrostatic forces, and a large contact angle change is not necessarily required for droplet movement, as illustrated in EWOD applications [4,5]. The thermodynamic approach however fails to explain the liquid-dielectrophoretic forces, which become predominant at relatively higher frequencies [17,31]. Under the influence of the external, non-uniform, electrostatic field (*E̅*), often imposed by patterned metal electrodes, a net dipole contribution (*P̅*) is generated inside the polarizable media, and the ponderomotive DEP force can be generally expressed as [39]:

(3) $$\bar{F}=\left({\bar{P}}_{\text{eff}}.\nabla \right)\bar{E}$$

**Figure 2.**
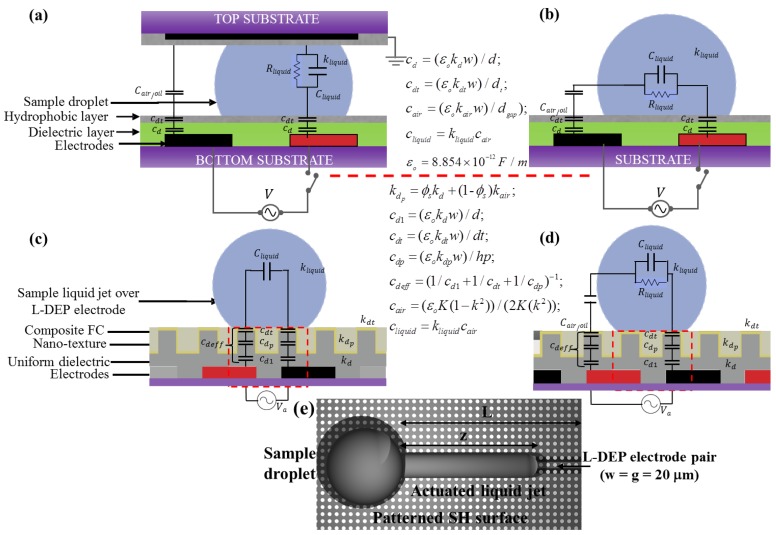
Electromechanical RC actuation model for: (**a**) dual surface, DMF droplet electro-actuation; (**b**) a single surface droplet electro-actuation; (**c**) L-DEP liquid jet actuation over coplanar electrodes and a superhydrophobic surface; (**d**) D-DEP actuation over a nano-textured superhydrophobic surface; and (**e**) schematic top view of an actuated liquid jet (L-DEP actuation).

The effective dipole moment (*P̅_eff_)*) in Equation (3) depends on the size, composition and geometry of the dielectric media. Estimation of the effective dipole moment and the field divergence is easier for simple fluids and electrode geometries, but the analysis become highly coupled for more than one and complex fluid compositions, actuated over different electrode geometries. A more suitable and general analysis for such cases is demonstrated by using a lumped electro-mechanical model where the driving electrical force is analysed based on an equivalent RC circuit [28,33,41]. Figure 2 shows a lumped RC circuit model for the different electro-actuation-based DMF methods. Using the lumped model to estimate the energy storage within the different capacitive layers and employing the principle of work for the lumped system, the DEP force can then be calculated. For example, for an L-DEP liquid jet actuation (in the z-direction) over a pair of coplanar electrodes, the driving DEP force can be estimated as [2,28,41];

(4) $${F_{\text{DEP}}=\frac{\partial W_{e}}{\partial z}|}_{V=\text{const}}=\frac{V^{2}}{2}\frac{dC(z)}{dz};W_{e}=C(z)\frac{V^{2}}{2}$$

where *W_e_* is the work done by the external electric field, *C(z)* is the lumped capacitance of the liquid propagating along the z-axis and *V* is the applied voltage. A similar approach has been reported and used to analyse the droplet actuation in EW-based systems [33].

The D-DEP actuation mechanism is a combination of these two electrostatic field effects, DEP and EWOD. The herringbone electrode pair, as shown in Figure 1d, is energized with a low voltage (∼100 Vrms) and low frequency (30–80 Hz) AC voltage, to induce periodic deformations in the droplet, which is placed at one end of the D-DEP electrode (see Figure 1d). It can be observed from the electrostatic simulation results that the resulting electric field will be very intense near the edges of the fishbone-shaped electrodes, which results in the meniscus of the droplet being dielectrophoretically pinned at the edge. The resulting deformation is a unidirectional, non-symmetrical one, causing a net periodic shift in the droplet as the AC electric field is applied, and as the field turns off, the pinned trailing edge of the droplet retracts, resulting in a net forward motion of the entire droplet. This periodic deformation takes place at twice the actuation frequency, resulting in the droplet being successfully transported [3,12,28].

**Figure 3.**
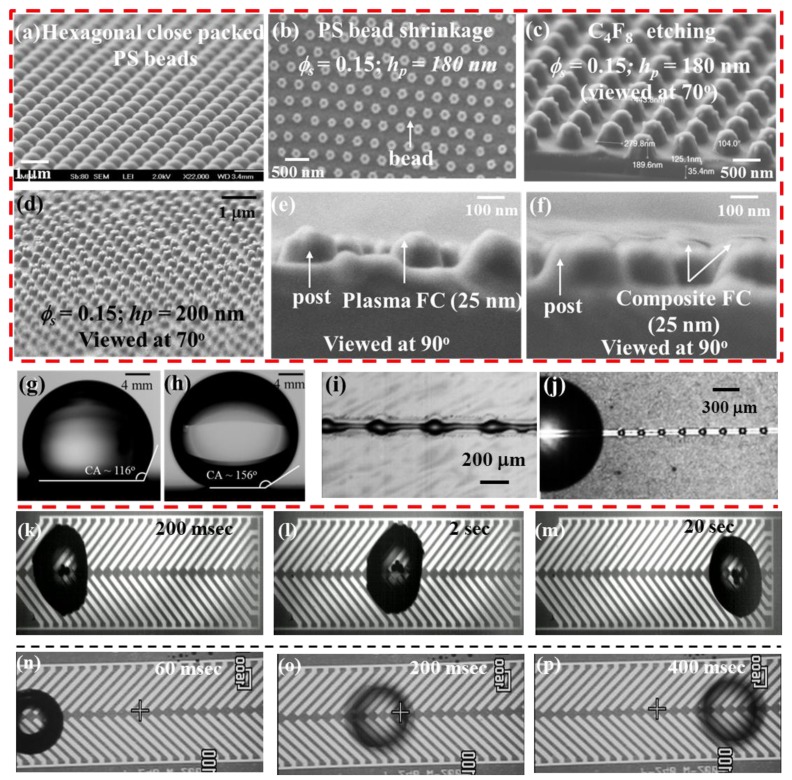
(**a**–**f**) Steps involved in the nano-texturing of a DMF micro-chip using soft-lithography (reproduced with permission from [49]); (**g,h**) a 5-μL de-ionized water droplet over a hydrophobic and a superhydrophobic (SH) surface; (**i,j**) micrograph showing the performance of L-DEP over a hydrophobic and a SH surface; (**k**–**m**) a 1-μL PCR droplet during D-DEP actuation over a hydrophobic surface; (**n**–**p**) a 1-μL PCR droplet during D-DEP over a SH surface. FC, fluorocarbon.

Figure 3 schematically shows periodic droplet motion during a typical D-DEP actuation. The frequency of actuation used for D-DEP actuation is restricted by the fluidic response (and hence, the fluidic damping) to the applied AC frequency. It was observed that at higher frequencies >150 Hz, an aqueous droplet is unable to resonate with the applied frequency, resulting in mere oscillations of the droplet while pinned at one specific point. In the case of D-DEP actuation under an oil bath, the actuation speed is further lowered due to viscous damping provided by the surrounding oil media. In such cases, successful D-DEP actuation can be achieved at low frequencies (∼50 Hz). Furthermore, the geometrical attributes of the D-DEP structure have to be carefully chosen based on the size of the daughter droplet that is to be actuated. The distribution of the electric field and DEP forces for a typical D-DEP electrode array, demonstrating the strong field regions and the periodicity in the field distribution along the centerline, was analyzed in [28]. The various DMF electro-actuation parameters and the range of fluidic parameters, suitable for these three droplet electro-actuation methods, have been previously reported in different studies [1,3,28] and are summarized here in Table 1.

## 3. Device Design and Fabrication Methods

DMF devices require a series of clean room fabrication processes to achieve the miniaturized dimensions and other structural requirements, such as: micro-patterned electrode architectures, dielectric insulation over the electrode structures and top hydrophobic or superhydrophobic surface topologies. A cross-sectional view of a typical micro-fabricated DMF device is shown in Figure 2. A majority of DMF devices are fabricated on silicon or glass substrates, as most fabrication facilities are well accustomed to handling such substrates. More recently, the use of polymers and other disposable substrates has been investigated for reducing the projected device cost in bio-diagnostic applications [34,36]. The use of a printed circuit board (PCB) as substrates is also becoming popular; especially for DMF devices dealing with microliter or larger droplet aliquots. The PCB substrates are advantageous because of their low cost printing and the possibility of integrating the wiring patterns and droplet actuation electrodes on a single substrate, through the use of the multilayer format of PCB substrates [36]. In more recent investigations regarding disposable DMF micro-chips, paper-based substrates have been micro-patterned using ink-jet printing techniques to print silver electrodes on custom paper-based substrates to form the bottom surface of the two-surface DMF micro-chip [37,38]. The DMF micro-electrodes are patterned in metals, such as aluminum, gold and chromium, or other conductive materials, such as ITO (indium tin oxide). The patterned electrodes are exploited to generate the required electrostatic field for droplet actuation. The micro-electrodes furthermore serve as open fluidic tracks over which the droplets are dispensed and transported (to different on-chip reaction sites), mixed and split.

Since most actuated liquids are aqueous samples of fairly high conductivity, it becomes mandatory to passivate the micro-electrodes with a thin dielectric layer (silicon nitride, silicon oxide, PDMS, SU8, *etc*.), in order to prevent sample electrolysis during liquid actuation. The final top surface of the device is then rendered hydrophobic by adding a thin layer of polymeric materials (typically parylene, Cytop™, silane and Teflon™) [28,41,42] or nano-patterned roughness can be utilized to create a superhydrophobic top surface (see Figure 3a–f). The superhydrophobic surface provides a very high droplet contact angle (>150°), which facilitates DMF droplet actuation and is especially useful for chip-based bio-assays, as discussed in the following section.

### Suitable Dielectrics and Surface Morphologies for DMF Devices

There are several crucial design issues to consider in implementing a reliable DMF device. The electrode structure is designed to provide the necessary electrostatic field effects; the insulating dielectric layer sustains the applied electric field while acting as a capacitive layer, which actively participates in the droplet manipulation, as illustrated in the electromechanical models of various DMF actuation schemes; and the top surface, which provides chemical inertness and fairly high contact angles (∼120° or more) for retaining the droplet shape during dispensing and other manipulation steps. Typical dielectric materials (Si_3_N_4_, SiO_2_, *etc*.) cannot provide all of the discussed properties to a DMF device. Although they are ideal as thin insulating layers and possess very high breakdown field strengths, they have poor top surface properties (very low droplet contact angle and high chemical affinity). Therefore, the dielectrics have to be used in combination with a thin hydrophobic top coating (e.g., Teflon AF™, Cytop™), which can be spin-coated on the top surface to overcome this limitation [28]. However, Teflon™ suffers from poor adhesion onto the Si_3_N_4_ layer, and limitations have been found for such combinations of a dielectric stack for repeated DMF actuations [52,53]. In order to improve this dielectric-Teflon™ adhesion, a multi-layered deposition method was developed in [52]. In this method, the deposited dielectric layer is cleaned using a very short span of plasma etching, which is followed by a plasma-enhanced deposition of a thin fluorocarbon (FC) layer, prior to spin-coating of the Teflon™ layer. The composite coating performs more robustly for the different electro-actuation-based DMF chips, as compared to a single spin-coated hydrophobic layer [52,53]. In some recent applications, the use of parylene-HT as a thin, hydrophobic, dielectric layer with a suitably high liquid contact angle has been demonstrated as an effective replacement for multi-layer dielectric stacks in electrowetting applications [54,55]. Parylene has also been utilized as a top layer for nano-textured, superhydrophobic surfaces [55]. In many applications, particularly when dealing with active enzymes and macro-molecules, the standard hydrophobic surface coating is not sufficiently reliable, as it is prone to sample adsorption and loss of the droplet contact angle, which hinders the diagnostic applicability of such devices. Several techniques have been used to minimize such adsorption issues [56], including the use of pluronics^®^, which are triblock copolymers of poly-(ethylene oxide) and poly-(propylene oxide) [57], and the use of a superhydrophobic top surface, which can be created by engineering nano-textures on top of the dielectric layer [49,58]. Both methods are effective at minimizing bio-adsorption; however, the use of pluronic^®^ is not a universal solution, since in several applications, such as PCR, pluronics^®^ can adversely affect the enzymatic activity during the reaction. Furthermore, the cost of nano-texturing can be significantly reduced by using a soft-lithography technique, as illustrated in [49] for DMF bio-applications.

As an example of a custom DMF microfabrication process, we illustrate the fabrication of the multiplex DMF PCR micro-device [21], which consists of: photolithographically-patterned chromium (Cr thickness: 200 nm) micro-heaters and resistance temperature detectors (RTDs) to create the thermostatic zones required for PCR thermal cycling; a photolithographically-patterned gold/chrome layer (100 nm Au/200 nm Cr) for electrical connections to the micro-heaters/RTD sensors; and another photolithographically-patterned aluminum (200 nm) layer for D-DEP and EWOD electrodes. These metal layers were electrically passivated with silicon nitride (∼500 nm), and the top dielectric surface was nano-textured into a superhydrophobic (SH) top surface, utilizing the method described in [49]. The SH surface provided a high droplet contact angle (CA ∼156°) during the device application and significantly reduces the extent of bio-sample adsorption.

## 4. Example Droplet Microfluidic Devices and Applications

Electro-actuation-based droplet microfluidics is a versatile liquid sample handling tool, which has been utilized in several bio-diagnostic applications. In this section, we review some example applications of DMF technology, which are categorized as: nucleic acid amplification and real-time detection; immunoassays; and protein analysis. Integration of suitable optical, electrochemical and other types of detectors is crucial for all of these diagnostic applications in order to provide accurate, error-free, real-time and quantitative assessment of bio-samples during the chip-based diagnostic procedure. Hence, the different optical detection methods and examples illustrating the integration of optics with DMF technology for such a bio-diagnostic application are also reviewed in the following section.

### 4.1. Nucleic Acid Amplification and Detection Assays

The handling and characterization of nucleic acid-based bio-samples are critical to many fields, such as: pharmaceutical research, clinical diagnostics and forensic studies. Nucleic acid-based bio-assays can be tailored to require lower sample/reagent volumes on miniaturized platforms, with high throughput and multiplexed screening capabilities. Hence, there has been a great deal of interest in the use of DMF for chip-based nucleic acid assays. In particular, DMF has been employed for the purification and extraction of nucleic acid samples [59–61], nucleic acid hybridization assays [52,62,63], PCR [18,20–22] and genomic sequencing [5,64]. Before a nucleic acid sample can be subjected to chip-based analysis, it must be extracted from complex matrix samples, which are collected from clinical patients. In one example of nucleic acid extraction using DMF, a DNA sample was purified from a complex matrix utilizing liquid-liquid extraction [61]. In this experiment, a droplet containing the nucleic acid and other proteins was driven in-and-out of a bath of phenolic oil that removed proteins from the droplet, hence purifying the nucleic acid. In another example, human genomic DNA was extracted from a whole-blood sample using an oil-filled two-plate DMF device by mixing droplets of whole blood with suitable lysis buffer [60]. The resulting droplets of cell lysate were combined with paramagnetic beads suitable for capturing DNA from the lysed sample and subsequently washed and eluted through controlled variation of ionic strengths of the two buffer solution droplets (washing and elution buffers).

PCR is a molecular diagnostic method used to greatly amplify target nucleic acids, extracted from clinical samples, in order to facilitate detection. Quantitative PCR (qPCR) facilitates real-time analysis and detection of specific nucleic acids during the amplification process and allows for quantitation of the target nucleic acid in the bio-sample. PCR at the microscale leads to a reduction of the reaction time, bio-sample and reagent volumes and the power requirement, due to faster reaction kinetics at such scales. The above capabilities can be achieved by utilizing microfluidic methods for manipulating nucleic acid samples and PCR reagents, either using continuous flow techniques or discrete droplets, cycled between two or more thermostatic temperature zones. Furthermore, a suitable fluorophore-labeled molecular probe is typically incorporated in the PCR reaction mix to facilitate real-time analysis of the PCR reaction. Since the early development of the conventional closed channel microfluidics-based PCR micro-devices, it has become evident that handling the PCR sample volume in the form of precisely dispensed, discrete microliter or smaller droplets results in improved performance of the PCR micro-device. The requirement of a high surfactant concentration in the continuous oil phase to stabilize the PCR droplets and the lack of individual addressing of the multiple droplets, along with the excessive need for off-chip plumbing are just a few challenging issues driving the development of miniaturized PCR set-ups towards DMF. In recent examples, DMF chips with embedded micro-heaters were designed to facilitate thermal cycling of PCR droplets [21,22,60]. In these studies, droplets containing c-DNA, which is to be amplified, and PCR reagents were merged, mixed and then thermally cycled between two or more thermostatic temperature zones. The efficiency of the chip-based quantitative PCR amplifications were comparable to those generated from bench-scale PCR equipment, and the total time and sample consumption were significantly reduced. In a recent example, chip-based PCR has been extended to the development of an automated, self-contained DMF platform, which can facilitate parallel sample preparation and multiplexed real-time PCR assays [20]. In this example, the DMF system has four cylindrical neodymium magnets for paramagnetic bead manipulation and four miniature fluorimeter modules consisting of four independent and spatially-separated channels for parallel PCR read-outs.

**Figure 4.**
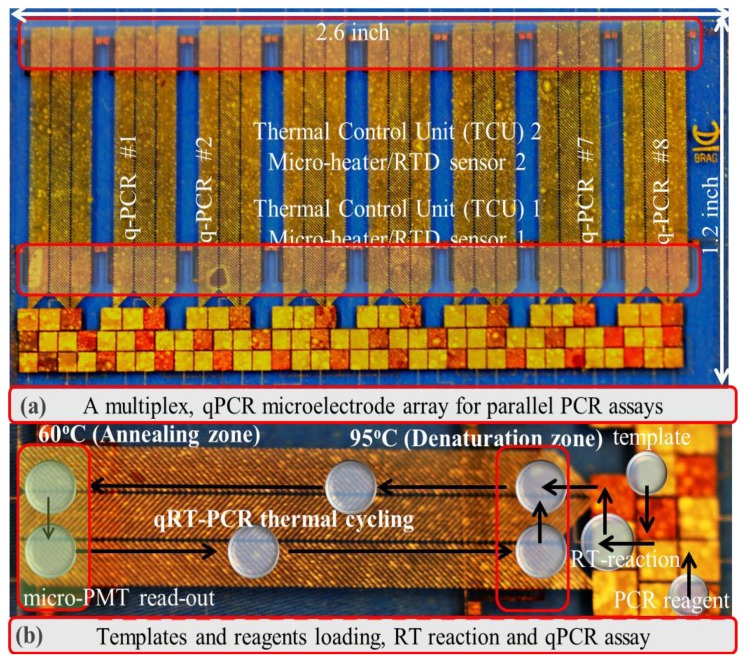
(**a**) Micrograph showing an eight-plex, parallel qPCR chip for panel PCR assays; (**b**) schematic illustration of the DMF chip-based single qRT-PCR assay system (reproduced with permission from [22]).

**Figure 5.**
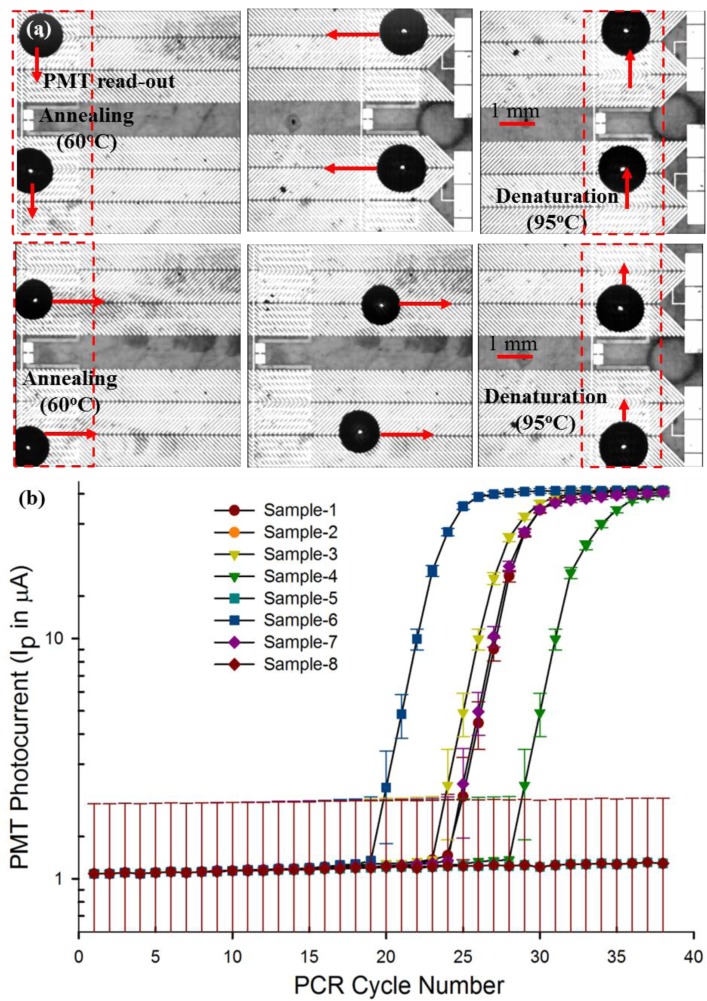
(**a**) Droplet-based parallel, real-time PCR illustrated using two 10-μL PCR droplets; (**b**) qPCR curves extracted from the parallel, multiplex PCR assay (reproduced with permission from [22]).

Our recent work has demonstrated that a continuous droplet transport scheme using D-DEP, which enables droplet transport and thermal cycling without the requirement of active electrode switching, would be a more effective solution for a PCR micro-device, since it substantially reduces the electrical overhead requirement needed in the aforementioned PCR chip examples, in order to realize a multiplexed qPCR system. We initially demonstrated a single qPCR micro-chip design, utilizing the droplet-DEP (D-DEP) electro-actuation method to achieve qPCR amplification of *in vitro* synthesized influenza viral RNA samples [21]. More recently, we have improved upon the previously designed continuous, D-DEP electrode architecture for droplet PCR thermal cycling, to reduce the cycling time and to extend the design towards a multiplex qPCR chip, which can conduct up to eight independently-controlled parallel qPCR assays on clinical panel samples (see Figure 4) [22]. Figure 5a shows the continuous, bi-directional actuation of two 10-μL PCR droplets as part of the eight-plex parallel, real-time RT-PCR reaction. The result of an example eight-plex parallel qPCR assay, conducted using a clinical panel sample of influenza A virus, is reported in Table 2. The outcome of the chip-based panel qPCR assay was verified with the commercial PCR set-up at the ProvLAB, Calgary (see Table 2). The chip-based quantification of influenza A viral RNA was achieved through the threshold cycle (*Ct*), extracted on-chip for the eight panel samples and compared to the measured *Ct* values at the ProvLAB, Calgary [22]. The various panel qRT-PCR experiments, conducted in this work [22], have confirmed that the multiplex PCR chip can successfully handle more than one target and markers to screen for a panel of viral/infectious diseases. The efficiency of chip-based qPCR assays was found to be reasonably within the accepted benchmark (qPCR chip efficiency ∼95%), and the completion time for 40 PCR cycles, including the 5 min of RT-reaction (reverse transcription reaction for converting viral RNA into DNA) with real-time multiplexed detection, for up to eight different PCR droplets was found to be 40 min. The multiplexed PCR chip was also used to amplify *in vitro* RNA samples, spiked over four orders of magnitude, to demonstrate the wide dynamic range and the low detection threshold of the developed DMF chip [22].

### 4.2. Immunoassays

**Figure 6.**
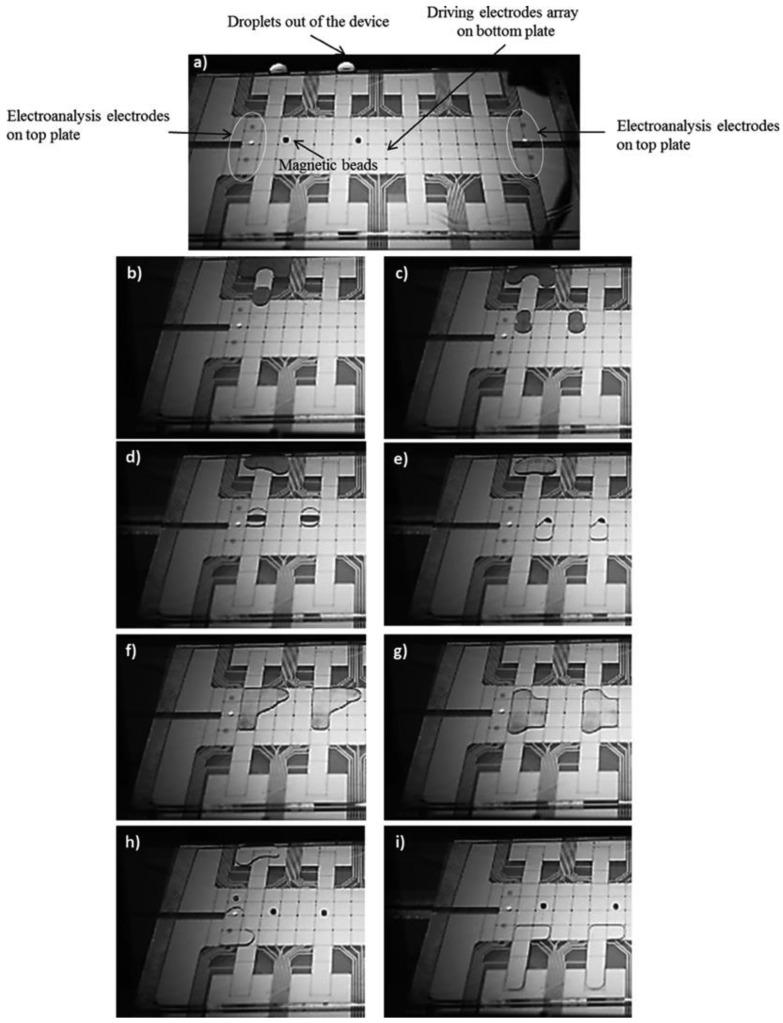
Micro-graphs showing an example immunoassay on a two-plate EWOD DMF chip (reproduced with permission from [23]);(**a**–**c**) dispensing and transport of magnetic bead, antibody and blocking protein mixture; (**d,e**) trapping of magnetic beads and splitting of unused solvent mixture using a magnet; (**f,g**) washing and resuspension of the functionalized beads; (**h,i**) elution of the captured antibody target from the magnetic beads and electro analysis using the top plate measurement electrodes.

Immunoassay is another molecular diagnostic technique that is often used for the detection of target macromolecules (proteins, antibodies), utilizing specific antibody-antigen bonding. In immunoassay techniques, a specific antibody is exploited to attach with target proteins through the antibody-antigen linkage, and the captured protein/macromolecule is concentrated to a detectable analyte concentration threshold [23,25]. There have been some very successful demonstrations of immunoassays on DMF micro-chips. Most of the DMF-based immunoassay experiments have relied upon custom antibodies, which were covalently linked to a solid surface, either on micro-beads [24–26] or a nano-textured solid surface [34], and these are termed as a heterogeneous immunoassay. One of the early DMF immunoassay examples for the detection and estimation of immunoglobulin G (IgG) and ricin used agglutination patterns of antibody-coated latex and gold particles [65]. In this approach, a droplet was suspended in fluorinated oil on a single-plate device with a controlled evaporation rate, and the pattern assumed by the antibody-coated latex and the gold particles as the droplet evaporated indicated the quantity of antigen present in the sample. This method reduced the sample volumes and limits of detection compared with commercially available methods, but produced a readout that may be difficult to standardize or automate for high-throughput analysis. In another example, an oil-filled DMF device was used to detect insulin and interleukin-6 by using droplets carrying paramagnetic beads modified with antibodies [23]. In another example, Nicolas *et al.* [66] have demonstrated a highly efficient collection and extraction of functionalized magnetic particles during an IgG immunoassay, on a DMF platform [66]. Figure 6 illustrates a general on-chip immunoassay protocol, which has been implemented in recent DMF immunoassay chips. A DMF chip-based heterogeneous immunoassay consists of the following stages:

- Mixing of droplets containing magnetic beads, reporter antibodies and blocking proteins with a droplet of analyte to form antibody-antigen complexes.
- The reaction mix being transported onto a target site, aligned with a permanent magnet to immobilize the magnetic beads.
- Removal of the excess supernatant by splitting the excess liquid from the beads via droplet splitting and/or by repeated washing steps using a wash buffer.
- Resuspension of the antibody from the magnetic beads into the elution buffer and read-out.

In the most common form of detection for immunoassays, the enzymes on the reporter antibodies are catalyzed with a chemiluminescent signal, which is detected by a CCD imager or an integrated photomultiplier tube. To demonstrate the clinical applicability of this DMF immunoassay scheme, a troponin I immunoassay was performed using whole-blood samples, where the authors reported efficient analyte recovery (77%–108%) [23]. In more recent experiments, researchers have explored the use of paramagnetic nanoparticles as a solid support for IgE immunoassays [34]. The authors demonstrated that nanoparticles can be dispensed by DMF with reproducible densities (CV, 2.83%), which is critical for any analytical application. In these IgE immunoassays, a droplet containing magnetic nanoparticles (modified with anti-IgE) and fluorescently-labeled IgE aptamer was merged and mixed with a second droplet containing a non-labeled IgE sample. Magnetic bead washing was performed in a similar manner as that described above. The assay was capable of detecting IgE at concentrations as low as 150 nM.

An alternative to the use of beads as the solid support for heterogeneous immunoassays is the direct immobilization of capture antibodies on the device surface. The first surface-based immunoassay on DMF, using human IgG as a model analyte, was demonstrated in [26]. In this study, the target antibodies were captured on top of a tailored hydrophobic surface of the DMF device, and the analyte concentration was detected using FITC (fluorescein isothiocyanate)-labeled anti-IgG. Pluronics F-127 was used as an additive to the assay solutions in order to prevent surface adsorption and contamination issues during the immunoassay.

### 4.3. Protein Analysis

Nucleic acid-based bio-diagnostic methods have exceled over the past few decades with enormous success in the form of micro-array and next-generation sequencing technologies; we have a much better grasp of human and other genomes. These genomic sequencing libraries have greatly assisted the current generation of nucleic acid diagnostic methods. PCR and its different versions are now the primary molecular diagnostic tool for the clinical detection of infectious diseases. Proteomics is another set of molecular bio-diagnostic techniques, where protein biomarkers are used to analyse human samples. A key challenge in protein-based bio-analysis is the need for rigorous and multistage sample processing to enhance the concentration levels of endogenic proteins and selectively target the low abundance protein variants, which can then be analysed through spectrometry-based detection. The attributes of DMF technology to individually address several reagents simultaneously and facilitate automated multiple stages of sample preparation makes it a viable choice for chip-based protein analysis.

**Figure 7.**
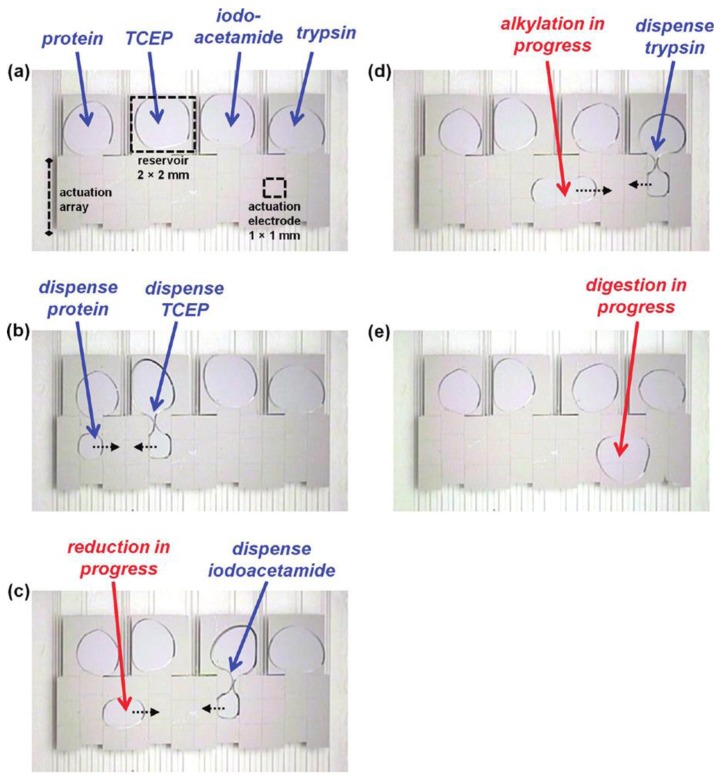
Protein analysis illustrated using a two-plate EWOD DMF chip (reproduced with permission from [66]); (**a**) pre-loaded samples/reagents on the proteomic chip; (**b,c**) protein reduction using tris-(2-carboxyethyl)phosphine (TCEP); (**d**) protein alkylation using iodoacetamide; and (**e**) the digestion step using trypsin.

In an early demonstration of protein analysis using DMF-based methods, purification of various peptide and protein samples from a heterogeneous mixtures was illustrated [67,68]. This included a series of steps, such as: drying of the sample droplet, rinsing of the dried spot with deionized water droplets to remove impurities and, finally, delivering a droplet of MALDI (matrix-assisted laser desorption ionization) markers to the purified proteins for on-chip analysis using mass spectrometry (MS) [68–71]. Recently, a DMF-based protocol was developed for extracting and purifying proteins from complex biological mixtures by precipitation, rinsing, and re-solubilization [72–75] (Figure 7). The method had protein recovery efficiencies (∼80%) comparable to those of conventional techniques and it required no off-chip sample processing, while minimizing the total processing time. In a separate study, DMF was applied to key proteomic processing steps that commonly follow protein extraction, including protein reduction, alkylation and digestion [73,74].

Peptide mixtures processed in this manner were analyzed off-chip by MALDI-MS and were identified by a Mascot database search engine, which yielded correct sample identification with a confidence level greater than 95%. In related research, on-chip protein biochemical processing was combined with *in situ* analysis by MALDI-MS [73].

In an alternative strategy, DMF has recently been used to deliver reagents to and from hydrogel discs [74], and this system has proven to be particularly powerful for forming heterogeneous micro-reactors for proteomic sample digestion. For a complete proteomic sample-to-detection assay protocol, an automated DMF-based platform was developed that demonstrated all of the commonly-used processes, including precipitation, rinsing, re-solubilisation, reduction, alkylation and digestion (Figure 7c–e).

## 5. Optics in Droplet Microfluidics

For any DMF-based diagnostic application, qualitative or quantitative analysis of the reaction either at the end point or in real-time during the reaction process is critical to the diagnostic component. DMF chips can be utilized for both in-line (droplets are analyzed on-chip with an integrated opto-electronic system) and off-line analysis (where post-assay aliquots are collected and analyzed away from the chip). The flexibility offered by DMF in the integration of more than one detector, both on- and off-chip, is not readily available in the competing closed channel microfluidic technology, which is often restricted to on-line, serial read-outs, such as flow cytometry. In this section, we look at some of the potential integrations of optical detection in DMF devices. It is also worth pointing out that optics in DMF technology has also been used as a feedback mechanism to precisely report the position of assay droplets during a reaction, giving the user unique control of different dynamic reaction parameters, such as temperature cycling time, washing and elution time periods, *etc*. [75,76]. Furthermore, the EW-based DMF technique has more recently been utilized in paper-based microfluidics with colorimetric detection [77].

**Figure 8.**
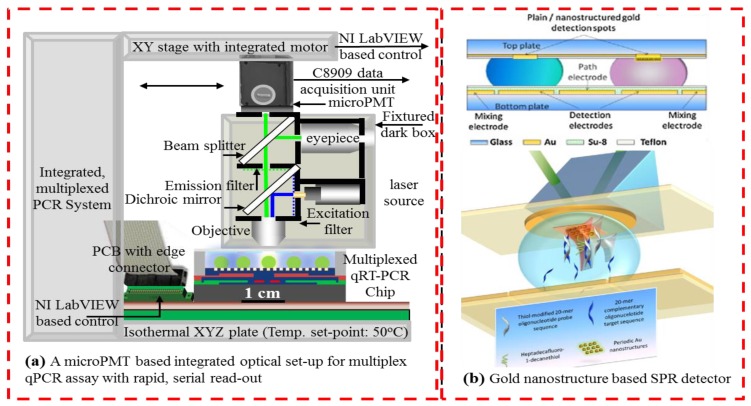
(**a**) Schematic diagram of an opto-microfluidic system with scanning micro photomultiplier tubes (μPMT) for multiplexed assay read-outs; (**b**) label-free detection using SPR and a nanostructured surface (reproduced with permission from [79]).

There are different optical techniques that have been leveraged for in-line or on-chip detection in DMF devices. In one example, a DMF system was reported, where absorbance in droplets was measured by integrating a light-emitting diode (LED)-based light source and a photodiode detector [78]. Analyte concentration during the liquid-liquid extraction process in droplets has been reportedly monitored using a light source and a CCD camera for image-based absorbance monitoring in [61]. On-chip absorbance-based detectors in DMF are often restricted by the available luminous intensity and the optical path to the assay droplet, and hence, fluorescence-based optical detection methods have been found to be more effective for the majority of DMF bio-applications. An example of fluorescence detection in DMF was demonstrated in [60] using a miniaturized fluorimeter, which was comprised of an LED and a photodiode. Photomultiplier tubes, or PMTs are another example of optical detectors that are very sensitive to changes in light intensity and are best suited for low light detection applications [21,22,63]. We have demonstrated the usage of a PMT set-up for reading up to eight parallel PCR reactions using a linear PMT scan, during the PCR annealing phase [22]. Miniaturized PMTs (μPMT, Hamamatsu) (Figure 8a) can be integrated with thin film wave-guides, fabricated right onto the DMF substrate and coupled with fiber-optics to provide an integrated optics-microfluidic system, which is compact, yet sensitive, for real-time, bio-diagnostic applications.

A semiconductor-based thin-film optical detector on a DMF device has also been utilized for chemiluminescence-based detection [80,81]. Electrochemical detection has also been integrated with DMF chips for inline analysis [82], and in an early demonstration, Dubois *et al.* [83] integrated a catenary wire for electrochemical detection in ionic reaction droplets. Surface plasmon resonance (SPR) has been demonstrated as a label-free detection method for chip-based diagnostic applications [62,79,84]. Such schemes utilize nano-textured surfaces, functionalized with specific bio-markers, which bind and immobilize specific target macro-molecules, resulting in a change in the optical path, which is detected through integrated optics. Malic *et al.* [79] coupled DMF with SPR imaging by using a gold layer deposited onto the top plate of their two-surface DMF microchip (see Figure 8b). Other label-free detection methods in DMF include: impedance spectroscopy [76], capacitive sensing [85–87] and field effect transistors [88] for label-free detection on an active chip surface.

Apart from the integration of in-line detectors, a modular approach where the detector can be an external, compact bench-top module, designed for rapid read-out upon loading the DMF chip, can be particularly effective for diagnostic laboratories where portability is not a key issue. There have been several examples of using off-line detection modules for DMF applications, such as: optical plate readers for absorbance [89], radiolabeling [90,91] and fluorescence [26,89] detection.

## 6. Conclusions/Future Outlook

In this review article, we have described several DMF actuation methods before focusing on electro-actuation-based DMF methods and their key applications. The theory behind DEP and EW droplet actuations and device fabrication methods has been briefly summarized. Various example applications of the electro-actuation-based DMF micro-devices for bio-diagnostics, using both synthetic and clinical samples, were reviewed. With the advent of disposable polymeric substrates, PCB-based devices and rapid prototyping techniques, DMF is becoming cost effective and more accessible for both clinical and research laboratories. The capabilities of DMF continue to improve as novel techniques for droplet actuation, detection and separation are adapted to such miniaturized platforms. DMF has demonstrated its advantageousness in many different fields, especially in the proteomics and nucleic acid-based bio-diagnostic applications, where complex samples can be pretreated and analyzed on a single device. Needless to say, the outlook of DMF technology looks to be very promising for clinical and other point-of-care application, which comes directly from its cost-effective fabrication and compact design, which offers palpable integration with miniaturized electro-optical components.

## Figures and Tables

**Table 1.** Actuation parameters and the range of fluidic parameters for different droplet electro-actuation methods.

**Actuation Method**	**Actuation Parameters**			**Fluidic Parameters**			**Actuation Droplet Volume (μL)**
	
	**Voltage (V)**	**Frequency (kHz)**	**Electrode Switching**	**Conductivity (mS/cm)**	**Viscosity (cSt)**	**Surface Tension (dyne/cm)**	
EWOD	10–100	0–20	Active	No limit	<10	>20	100 μL–100 nL
L-DEP	100–400	30–500	N.A.	Frequency dependant (few mS/cm)	<10	>20	1 μL–10 pL
D-DEP	50–150	0.03–0.10	N.A.	No limit	<5	>40	100 μL–100 pL

**Table 2.** Details of the influenza A clinical panel exploited during the multiplex qPCR assay.

**Sample No.**	**Sample Type**	**ProvLAB *Ct***	**Chip *Ct***
1	Nasopharyngeal swab	29	25
2	Nasopharyngeal swab	Negative	Negative
3	Throat swab	30	26
4	Nasopharyngeal swab	32	30
5	Nasopharyngeal swab	Negative	Negative
6	Nasopharyngeal swab	24	20
7 (positive control)	H3 M-gene *in vitro* RNA	29	26
8 (negative control)	PCR water	Negative	Negative
